# Recurrent network dynamics reconciles visual motion segmentation and integration

**DOI:** 10.1038/s41598-017-11373-z

**Published:** 2017-09-12

**Authors:** N. V. Kartheek Medathati, James Rankin, Andrew I. Meso, Pierre Kornprobst, Guillaume S. Masson

**Affiliations:** 1 Université Côte d’Azur, Inria, Biovision team, Sophia Antipolis, France; 2 College of Engineering, Mathematics and Physical Sciences, University of Exeter, Exeter, UK; 3 Center for Neural Science, New York University, New York, USA; 4 Institut de Neurosciences de la Timone, CNRS and Aix-Marseille Université, Marseille, France; 5 Psychology, Faculty of Science and Technology, Bournemouth University, Bournemouth, UK

## Abstract

In sensory systems, a range of computational rules are presumed to be implemented by neuronal subpopulations with different tuning functions. For instance, in primate cortical area MT, different classes of direction-selective cells have been identified and related either to motion integration, segmentation or transparency. Still, how such different tuning properties are constructed is unclear. The dominant theoretical viewpoint based on ﻿a linear-nonlinear feed-forward cascade does not account for their complex temporal dynamics and their versatility when facing different input statistics. Here, we demonstrate that a recurrent network model of visual motion processing can reconcile these different properties. Using a ring network, we show how excitatory and inhibitory interactions can implement different computational rules such as vector averaging, winner-take-all or superposition. The model also captures ordered temporal transitions between these behaviors. In particular, depending on the inhibition regime the network can switch from motion integration to segmentation, thus being able to compute either a single pattern motion or to superpose multiple inputs as in motion transparency. We thus demonstrate that recurrent architectures can adaptively give rise to different cortical computational regimes depending upon the input statistics, from sensory flow integration to segmentation.

## Introduction

Sensory inflows received by animals are highly complex and ambiguous with multiple local sensory events occurring simultaneously. A challenging computational task faced then by any sensory system is to either integrate or segment these local signals in order to encode behaviorally relevant information. This is well illustrated by visual motion processing. Local motion signals must be selectively integrated in order to extract and reconstruct the direction and speed of a particular surface^1^. But the same set of local signals must also be kept segregated from the many others belonging to distinct surfaces that can be either adjacent (e.g., motion boundaries) or overlapping (e.g., motion transparency). The rules governing motion integration and segmentation have been extensively investigated at both perceptual and physiological levels (see refs 2–4 for reviews). For instance, when presented with two motion directions, the primate visual motion system can group them according to linear (i.e., vector average) or nonlinear (i.e., intersection-of-constraints) rules^5, 6^. It can also segment them by either suppressing one of the two inputs (i.e., winner-take-all) or simultaneously representing both of them (i.e. superposition) as in the challenging case of motion transparency^7–9^.

In the monkey middle-temporal (MT) cortical area, a pivotal processing stage for object motion computation^4^, different classes of direction-selective neurons have been linked to each of the aforementioned computational rules (e.g. refs 6–12). When presented with moving plaids made of two superimposed sinusoidal gratings drifting in different directions, some cells encode only one of the two components (i.e. component cells) while others encode the pattern motion direction after combining them (i.e. pattern cells)^6, 12^. Recently, Xiao and Huang^10^ investigated the responses properties of MT cells when tested with bi-directional random dot patterns. A majority of MT cells exhibit a single peak tuned to either one of the components or to their mean direction, implementing winner-take-all or vector average computations, respectively. Other cells show two peaks and could thus represent two overlapping motion directions^10^. At the population level, a similar set of canonical tuning curves were reported and ascribed either to motion integration, motion segmentation or motion transparency, respectively^7, 10, 12^.

Several modeling studies have suggested that these subpopulations span a continuum along which their feed-forward, direction-selective inputs from area V1 are differently weighted^13, 14^. There are however several pitfalls with linear-nonlinear (LN) feed-forward models. First, their static nonlinearities cannot render the complex temporal dynamics of these tuning functions that were shown to shift over time from, say vector average to either winner-take-all or transparency solutions^9, 10, 15, 16^. Second, they cannot explain why exchanging plaid patterns by random-dot patterns with similar motion component directions scrambles the cell’s classification, nor can they explain contradictory results reported when attempting to predict MT cell tuning to one stimulus from the other^10, 11^. An alternative approach would be considering sensory integration and segmentation as threads of a complex dynamical computation where inhibition and excitation are shaped adaptively to the spatiotemporal properties of the inputs^17^. Only a handful of computational studies have investigated the interest of recurrent networks in visual motion processing. They succeed in explaining some properties of MT neuronal tuning functions, but have so far focused on only a small subsample of tuning classes and motion inputs^18–23^. Here, we designed a neural field dynamical model working in a motion direction space and investigated the interplay between its connectivity and input properties. We show that the different MT neuronal classes reported previously are steady-state solutions of the dynamical system, their strength and stability varying with both input statistics and excitation-inhibition balance. In specific parameter regions, the network exhibits coexisting states, leading to multi-stability across trials without adaptation mechanisms. We show how slightly asymmetric inputs can stabilize the network behavior. The temporal dynamics of direction tuning and the transitions between different network states when varying input statistics are also well captured. These results demonstrate a fundamental role for recurrent connections in shaping basic cortical computations and show that sensory neural circuits can dynamically switch from integration to segmentation depending on input statistics.

## Results

We studied the behavior of a nonlinear voltage-based network model that describes the local mean field potential (see Methods) of a population of directionally tuned MT neurons under different inputs and excitation-inhibition interactions. We focused on the properties of the steady state solutions such as the shape of the direction tuning functions, their number of peaks at convergence and the peak positions with respect to the driving inputs. A recent and comprehensive physiological study of MT responses to different types of uni- or bi-directional motion stimuli^10^ provides an ideal point of comparison for our model simulations.

**Figure 1:**
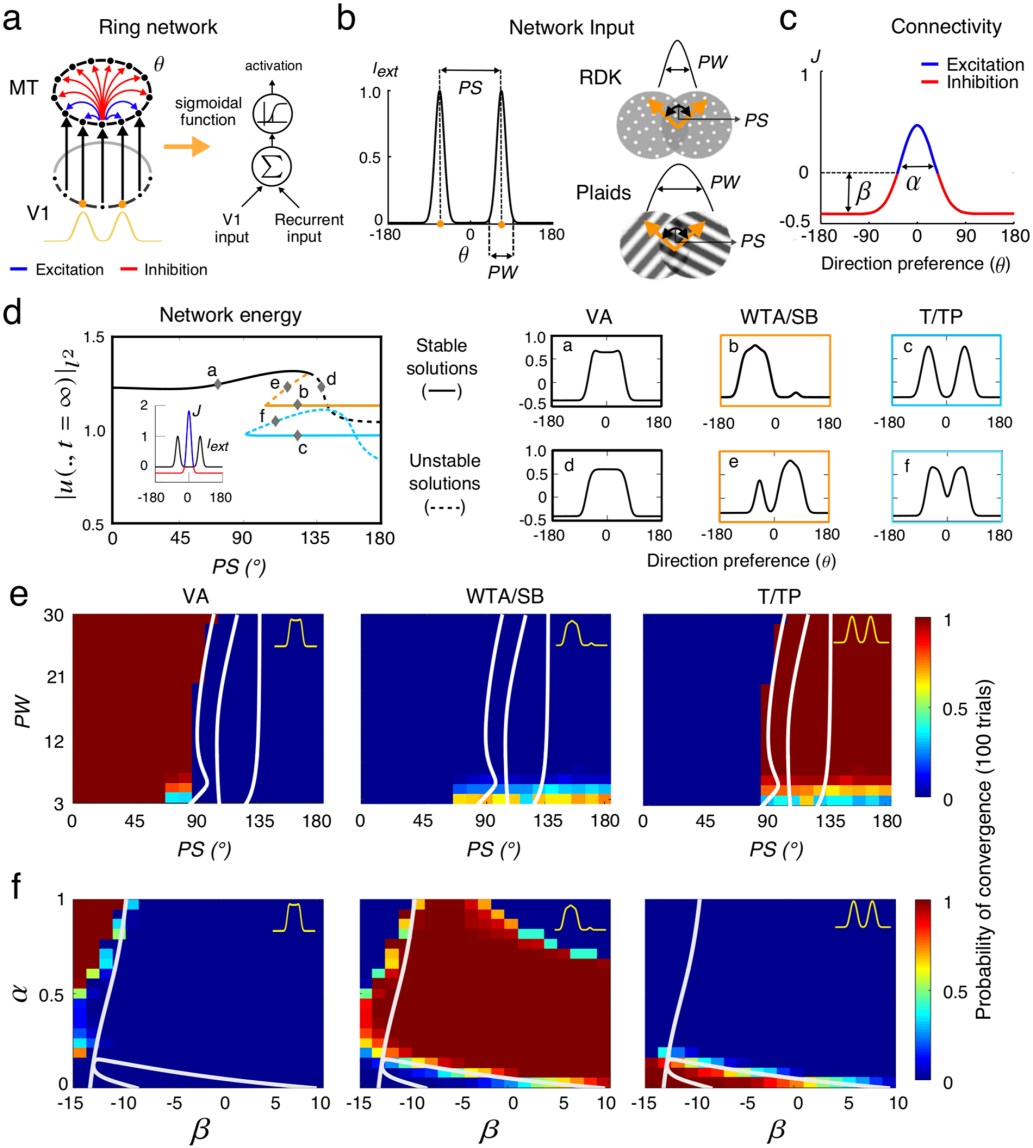
*Ring model behavior and likelihood of convergence*. **(a)** Illustration of the ring network modeling visual motion integration at MT cells level, with both V1 input and recurrent connections. **(b)** MT inputs are defined as two Gaussian distributions with parameters *PS* and *PW*, corresponding to different random dot and plaid patterns. **(c)** The center-surround connectivity kernel in motion direction space is set by parameters α and β, whose combinations defined a family of connectivity kernels. **(d)** Network energy is computed from the I2-norm of the steady-state solution. The bifurcation diagram is plotted as a function of parameter *PS* (with *PW* = 10°, α = 10 and β = −10, fixed values) to illustrate both stable (*a*, *b*, *c*) and unstable (*d*, *e*) solutions for each branch and their corresponding direction tuning functions. Three classes of solutions co-exist: vector average (VA), winner-take-all (WTA) and transparency (T). Note that referring to^10^, winner-take-all will also be designated as side-biased (SB) and transparency as two-peaked (TP) tuning curves. **(e)** and **(f)** show attractor strength of each steady-state solutions (*a*, *b*, *c*), measured as a probability of reaching a particular solution from 100 repeated simulations done with initial random conditions, and varying either input parameters (then fixing α = 0 and β = −10) or connectivity parameters (with fixed *PS* = 120 and *PW* = 10), respectively.

The network represents a sub-population of *N* directionally tuned MT neurons with a smoothly varying directional preference, represented by an angle θ (Fig. 1a) consistent with MT direction bandwidths previously reported (see ref. 24). Each cell receives afferents from an input layer, where motion stimuli are encoded by Gaussian distributions in direction space similar to V1 direction selectivity (Fig. 1b). In the current study, we specifically investigated the processing of bi-directional motion stimuli such as plaids or random dot patterns. These inputs can be equally well described as the sum of two Gaussian distributions. The MT input is thus defined by the peak width of each Gaussian distribution (*PW*) and the peak separation between the two distributions (*PS*) in order to simulate different types of bidirectional motion stimuli. Distributions are broader for gratings than for random dot patterns, reflecting the larger inherent ambiguity of grating motion direction relative to random dot patterns. This broadening is also consistent with the fact that both V1 and MT cells respond with wider direction and speed tuning to gratings^24–26^. Lastly, MT neurons also receive inputs from the local recurrent interactions (Fig. 1c). This local recurrent connectivity depends only on the directional difference, being locally excitatory and laterally inhibitory. It implements a typical center-surround connectivity kernel defined by a weighted difference between Excitation and Inhibition Gaussians kernels in the motion direction space. Note that we consider here the limiting case of global lateral inhibition (i.e. a very broad inhibitory Gaussian kernel). Such simpler architecture would preserve higher order harmonics rather than reducing to a two^27^ or three modes approximation^28, 29^. The connectivity kernel is defined by only two parameters: the extent in direction space of lateral excitation (α) and the strength of inhibition (β) (Fig. 1c).

### Network behavior

Using numerical bifurcation analysis, we first identified the possible stable solutions and the parameter regimes over which different solutions coexist (see Table 1 for initial network parameter settings). When the network is stimulated with a bidirectional input, varying *PS* leads to different types of solutions. These are shown in a bifurcation diagram in Fig. 1d, where solid and dashed curves represent stable and unstable solution branches, respectively. The tuning curves of the three stable solutions are illustrated in the right-hand panels and corresponded to the three main neuronal tuning reported by^10^. Cases *a*, *b* and *c* in Fig. 1d correspond to the observed vector average (VA), winner-take-all (WTA) and transparency (T) tuning functions, respectively. For small *PS*, VA is the only possible solution. For large *PS*, WTA and T coexist. For an intermediate range (95° < *PS* < 130°), we identify a critical operating regime where the model is capable of producing all three prototypical tuning functions within the same parameter region. We observed that the WTA solution was side-biased (SB), indicating that the other, competing input was not fully suppressed, similar to the empirical evidence reported in macaque area MT^10^. Unstable solution branches (*d*, *e*) can link the stable branches, but are not critical in this study. Stable solution branches terminate at bifurcations points where a qualitative change in model behavior occurs. We note that the intersection of (*a*, *d*) is a pitchfork bifurcation (with two complementary WTA branches overlapping in the projection shown) while intersections of (*b*, *d*) and (*c*, *e*) are fold bifurcations^30^. Two unstable branches overlap in this projection at *PS~145°* but are not connected. These can be tracked in terms of a two parameter phase diagram, delineating entire parameter regions where different types of solutions exist (see continuous white lines in Fig. 1e,f.

**Table 1:** Model parameters and simulation values.

Description	Parameters	Value
Number of sample in [−π, π]	N	404
Sigmoid threshold	th	3.0
Sigmoid gain	μ	16.0
Input gain	κ_ι_	0.1
Population time constant	τ_p_	1.0, 5.0, 10.0 ms
Inhibition time constant	τ_l_	30–100 ms
Homotopy variable to regulate excitation width	α	[0,1]
Inhibition offset	β	[−10,15]
Excitation width	${\sigma }_{{e}_{\alpha }}={\sigma }_{{e}_{a}}+\alpha ({\sigma }_{{e}_{b}}-{\sigma }_{{e}_{a}})$	$[11.5^\circ ({\sigma }_{{e}_{a}}),\,60^\circ ({\sigma }_{{e}_{b}})]$
Inhibition width	σ_i_	1800 ($\gg$360)
Excitation strength	${g}_{{e}_{\alpha }}$	${e}^{\frac{-{\sigma }_{{e}_{\alpha }}^{2}}{2}}$
Inhibition strength	${g}_{{i}_{\alpha }}$	$\frac{1+{g}_{{e}_{\alpha }}}{0.0797}$
Input Peak separation	PS	(0°,180°]
Input Peak width	PW	5°–30°

The network behavior is best characterized by maps of attractor strength that unveil which of the stable solutions identified in the bifurcation analysis dominates. Attractor strength of each solution, measured as a probability of reaching it from 100 repeated simulations with randomized initial conditions, was computed at every combination of both external (input: *PS*, *PW*) and internal (connectivity kernel: α, β) parameters (color maps in Fig. 1e,f). Although in a given parameter regime, two or more direction tuning shapes can coexist as stable solutions, the network might be much more likely to converge to one of these from a random initialization. In Fig. 1e, the likelihood of the VA solution was maximal for small *PS*, regardless of the PW (see left panel). For large *PS*, the transparency case dominates over a large range of PW (see right panel). For narrow (small *PW*) and widely (large *PS*) separated peaks the WTA solution occurs in roughly 60% of trials and TP otherwise (see middle panel). In Fig. 1f, we observe that maintaining two peaks at population level requires both low inhibition and narrow excitation (small β and α, see right panel) whereas VA requires low inhibition but broad excitation (small β and large α, see left panel). The WTA solution is highly probable across a wide range of inhibition strength and excitation extent. Thus, the emergence of different network states corresponding to either motion integration or segmentation results from the interplay between the properties of the inputs and the shape of the center-surround interactions in direction space. We propose that different properties of lateral connections are part of what distinguishes observed cell classes. In the following sections, we investigate the dynamics of these cell classes, focusing successively on the relationships between single-cell and population tuning, the stability of direction tuning and its temporal dynamics and the shift from one solution to another as a function of input statistics.

### Local recurrent interactions lead to prototypical tuning behaviors found in macaque area MT

**Figure 2:**
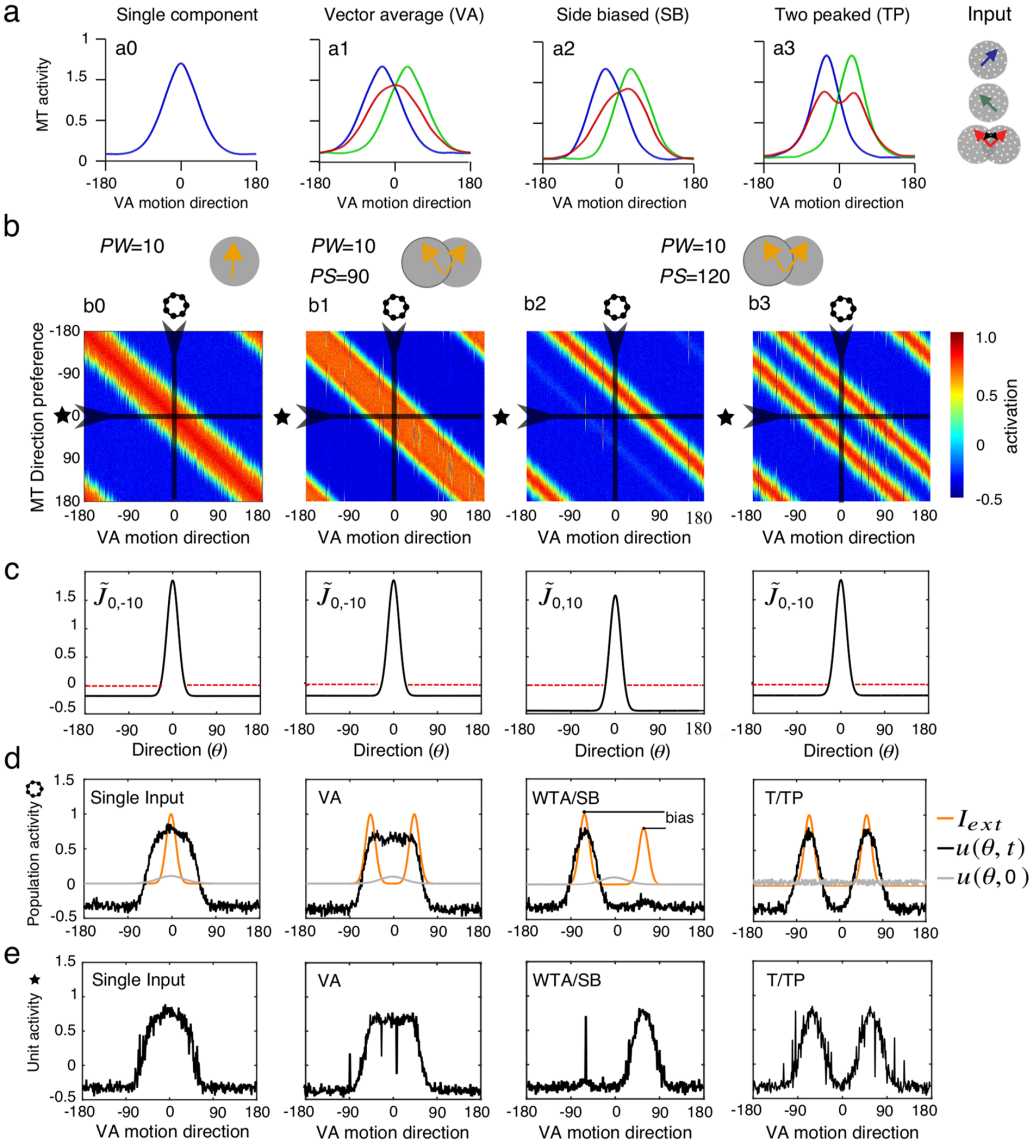
*Recurrent interactions lead to prototypical tuning behaviors found in MT*. **(a)** Four examples of prototypical single cell tuning functions found in macaque area MT (adapted from ref. 10). Blue and green curves show the tuning functions obtained when presented with a dot pattern moving coherently in one of the two directions. Red curves show the tuning functions obtained when the two direction components overlapped, forming a bidirectional moving pattern. For comparison, the thin blue curves are the predicted vector average profile from the single input tuning functions**. (b)** We simulated the ring network by sequentially varying the pattern direction of the driving input for a uni-directional stimulus (column b0) or different bi-directional patterns (columns *b1*–*b3*). The connectivity kernels used for each set of simulations are indicated in **(c)**. The orange curves illustrate the network inputs. **(d)** The tuning function of the MT population (indicated by a ring) can be obtained by cutting a vertical slice in plot b. Conversely, a single-cell tuning (indicated by a star) is obtained from an horizontal slice and shown in **(e)**. Notice that for column *b2*, single-cell and population tuning are symmetrical as we conserve the same sampling direction across all *b0*–*b3* conditions.

In macaque area MT, Xiao and Huang^10^ extensively documented the single-unit responses to random dot patterns made of either one or two motion direction components. For single motion inputs, direction tuning resembles the classical Gaussian-like functions as shown in panel *a0* in Fig. 2a. Using overlapping motion inputs with different degrees of component separation, they reported a handful of prototypical behaviors reproduced in Fig. 2a (a1 to a4, see Fig. 4 in ref. 10 **)**. For a fixed PW and two direction differences (PS), they described three different classes of cells. Type *a1* (*PS* = 45°) represents the vector average (VA) of the two motion inputs while *a3* (*PS* = 135°) superposes them, yielding to a two peaked tuning function (TP). Type a2 (*PS* = 120°) and its mirror symmetric (not shown) form a single class where one or the other of the two inputs is suppressed. These side-biased (SB) cells implement a weak winner-take-all where an influence from the suppressed inputs can still be seen. We show below that a population of direction selective cells can produce these prototypical behaviors through the influence of local recurrent interactions.

**Figure 3:**
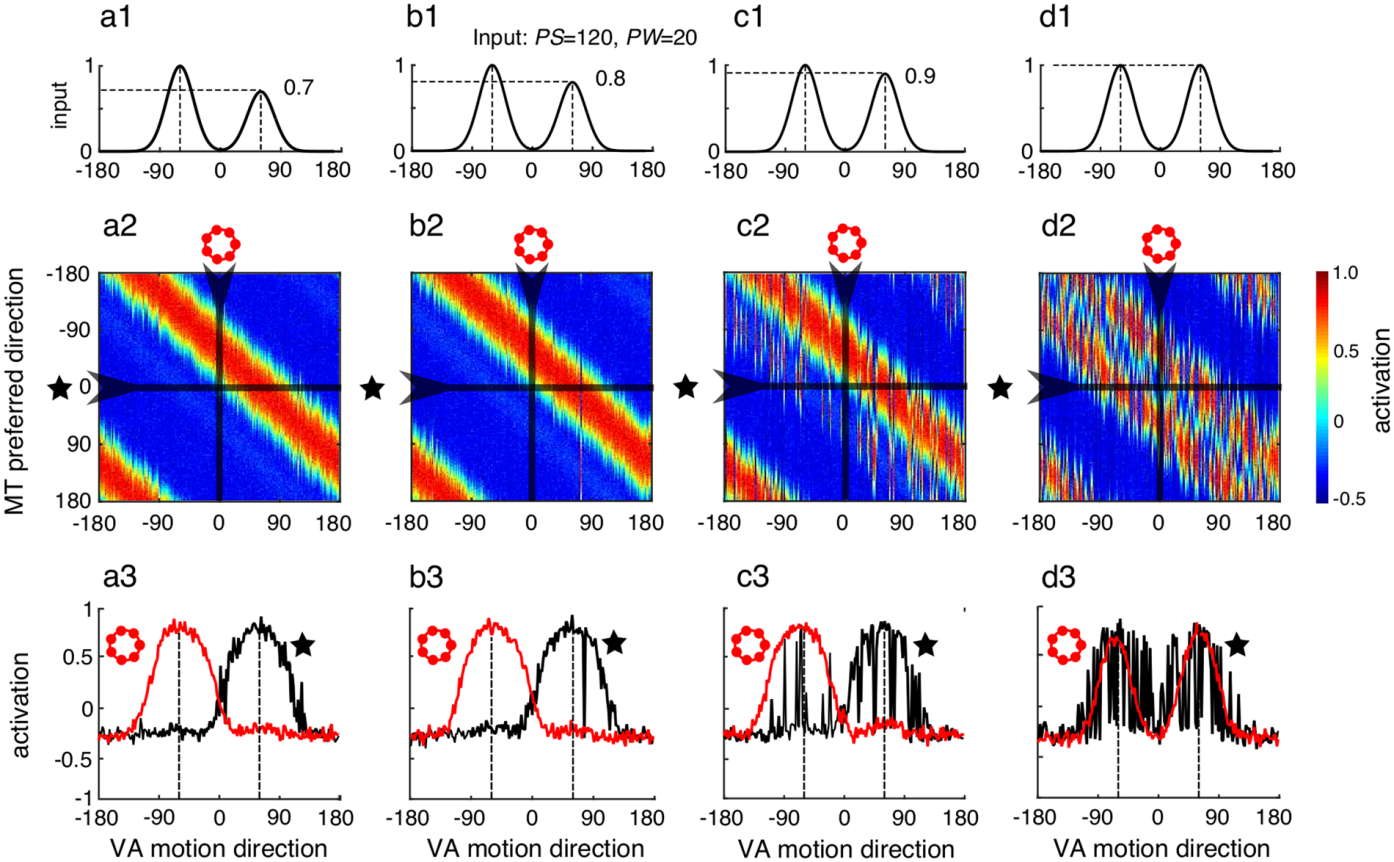
*Tuning behavior changes with respect to the asymmetry in the input*. We simulated the network with a single set of driving input directions (*PS* = 120°, *PW* = 20°) and recurrent inhibition parameters (α = 0, β = −10). From left- to right-hand plots, the relative strength between the two inputs increases from 0.7 to 1. Upper row illustrates the network inputs. Middle row shows the model responses as a function of both VA motion direction and population preferred direction. Again, vertical sampling of the response map gives the population tuning (ring) while horizontal sampling (star symbol) provides the single-cell tuning. Population (red curves) and single-cell (black curves) tuning functions are then plotted against VA direction in the lower row.

We simulated the network with a set of input pattern directions (see *Methods*). Figure 2b illustrates the MT population direction preference, as a function of the average input motion direction. Four cases are illustrated, corresponding to different recurrent connectivity regimes (Fig. 2c) and to different input parameters (*PW* and *PS* given in panel b and plotted as orange curves in Fig. 2d. Sampling the population vertically gives the MT population tuning (plotted in Fig. 2d) and sampling horizontally gives the single cell tuning (plotted in Fig. 2e). Note that with the lumped excitation-inhibition description used in our model, positive responses indicate motion directions with net excitation and negative responses those with net inhibition. Column *b0* shows that the network accurately represents the unidirectional input. Column *b3* illustrates the population and single cell tuning for two widely separated inputs (120°) and the same recurrent connectivity, with a low inhibition regime (β = −10). The two peaks are preserved, yielding to a bimodal tuning function (T/TP). Notice that each peak is now sharper than observed with a single direction input, thanks to recurrent inhibition. Narrowing the input direction difference to 90° (column *b1*) changed the tuning functions at both population and single-cell levels: now the vector average (VA) is represented. Using the same input as in *b3*, column *b2* illustrates that the network shifted to a winner-take-all solution when inhibition strength was increased (β = 10). Notice that, to favor the existence of a small second peak, we introduced a small input bias in favor of the −60° direction (Fig. 2d, column b3, orange curve) because a high inhibition regime together with a small bias within the input strengths leads to a robust side-biased (WTA/SB) behavior. Indeed, a small response to the suppressed direction is still evident, due to the local recurrent excitation, in both the population and single-unit tunings. We will further compare symmetric and asymmetric inputs below (see Fig. 3). Overall, by comparing biological (*a0-a4*) and simulated (*b0-b4*) responses one can clearly see that the ring model can reproduce the three prototypical tuning functions observed in macaque area MT (e.g. refs 10 and 11). Note that model single cell selectivity can exhibit variability due to recurrent interactions. This can happen when the network is operating in a parameter regime where different solutions co-exist with one attractor largely (but not totally) dominating. In this case, noise in the input can drive the network to different stable solutions from one simulation to the next due to the coexisting states shown in Fig. 1d. We will further investigate this below.

### Fluctuations in component selection and population tuning reliability

One of the hallmarks of recurrent interactions is the existence of multiple stable solutions for the same set of parameters. Under such multi-stability, the network could converge to different stable states (with different direction tuning) from one trial to the next. This is evident from the bifurcation diagram shown in Fig. 1d where different solutions can coexist for a given input (e.g., see at *PS* = 120°). Moreover, in the WTA regime, the network could select either of the two motion components when stimulated with inputs having purely symmetric component strength. Still, a stable tuning behavior could emerge in regimes where only one of the tuning behaviors is dominant, due to either inhibition strength or the structure of the driving input. To probe network stability, we fixed the recurrent connectivity parameters and used random dot patterns (*PW* = 20°) with a constant *PS* (120°) while varying the relative strength between the two motion components from 0.7 to 1 (Fig. 3, left-to-right columns). In empirical studies, the strength of one component can be reduced by decreasing its signal-to-noise ratio. For each condition, we ran 400 trials by sequentially shifting the vector average motion direction from −180 to 180°. The MT preferred direction is plotted as a color map similar to Fig. 2b. Under these conditions, WTA solutions dominate the network dynamics when the two inputs have different strength (plots a–c). The lower row shows stable tuning functions for both the population activity (red curves, lower row) and the sampled single-unit activity (black curve). The population activity matches the direction of the stronger input. When increasing the relative input strength to 0.9 (case c), the population response shifted from a pure WTA to a SB tuning function (see small bump at 60° in red curve of Fig. 3c, lower row). Now, single unit activities exhibited large fluctuations across directions, the sampled cell representing either one peak (i.e. SB tuning) or two peaks (i.e. T/TP tuning), with a strong dominance for the former. Hence, under this low inhibition regime (α = 0, β = −10), the effective inputs must be slightly asymmetrical to produce the SB solution. With symmetric inputs (Fig. 3d), the two inputs yield a shift in both population and single-unit tuning, now largely representing the two inputs (TP tuning) but with some occurrence of the WTA solution. Note that the single-unit activity was now largely fluctuating across the different VA motion directions (Fig. 3d, bottom row, black curve). As a consequence, with symmetrical inputs the ensemble of single-unit activities was much less consistent when the TP behavior was observed at the population level (compare left- and right-hand columns in Fig. 3). We conducted the same analysis for other *PS* and *PW* values and obtained similar results: population tuning properties changed and were less consistent when inputs were strictly symmetrical.

### Temporal dynamics

**Figure 4:**
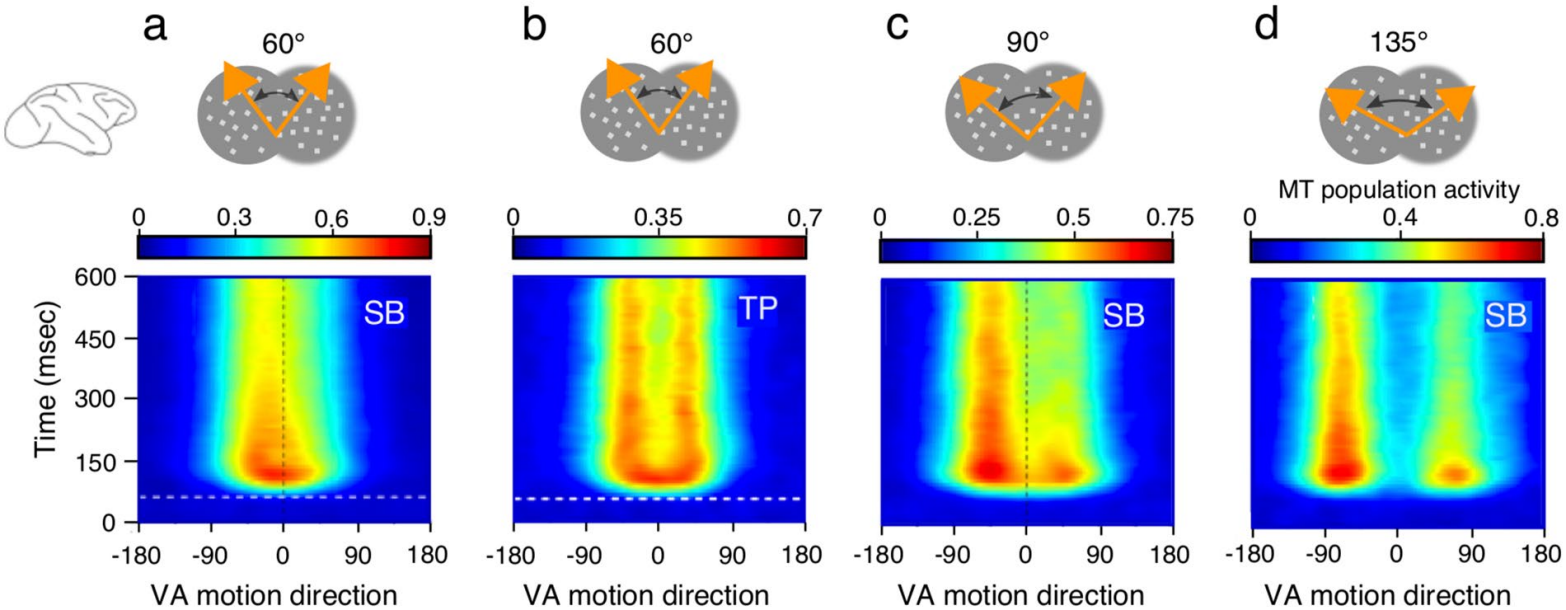
*Four examples of temporal evolution of MT tuning functions*. Panels **(a**–**d)** show the temporal development of the subpopulation-averaged response tuning to bidirectional stimuli with different *PS* values (adapted from ref. 10). **(a)** The initial response tuning peaks around the VA direction before gradually shifting to one single peak aligned with the population preferred direction, corresponding to the WTA solution. **(b)** For the same *PS* (60°), the two component motion directions gradually emerge, together with a strong suppression of the VA direction. **(c)** With *PS* = 90°, the initial response is a broad tuning centered around the VA direction before shifting towards the component motion aligned with the preferred-direction. **(d)** With larger *PS* (135°), the initial response tuning show two peaks (TP) before one peak is suppressed, leading to the SB tuning described above. All examples are adapted from Figs 6 and 8 of ref. 10, respectively.

Several empirical studies have shown that MT neuronal selectivity gradually develops over time after a latency of 50–70 ms^10, 15, 16^. Such temporal dynamics is well illustrated in the four examples taken from Xiao and Huang^10^ and re-plotted in Fig. 4 for different PS values. The examples in panels a-d show that steady-state WTA/SB tuning functions can gradually emerge from different early tuning shapes such as VA (*a*), SB (*c*) or TP (*d*). In case (*b*), this particular cell first encodes the vector average direction (VA) of a random dot pattern with a (60°) direction difference. The response tuning then gradually evolves towards the steady-state T/TP attractor solution in our model, where the two inputs are superimposed. By comparing these response patterns with the linear prediction from neuronal responses to each of the two components presented alone, the authors^10^ suggested that VA motion direction was suppressed while component motions were facilitated. This observation is consistent with the widespread hypothesis that lateral excitatory interactions without inhibition could lead to early integration with a broadly tuned response. Later on, neuronal response could be further shaped by the growing inhibition until they reach a stable solution that depends on the input statistics (e.g. refs 9, 10 and 31).

**Figure 5:**
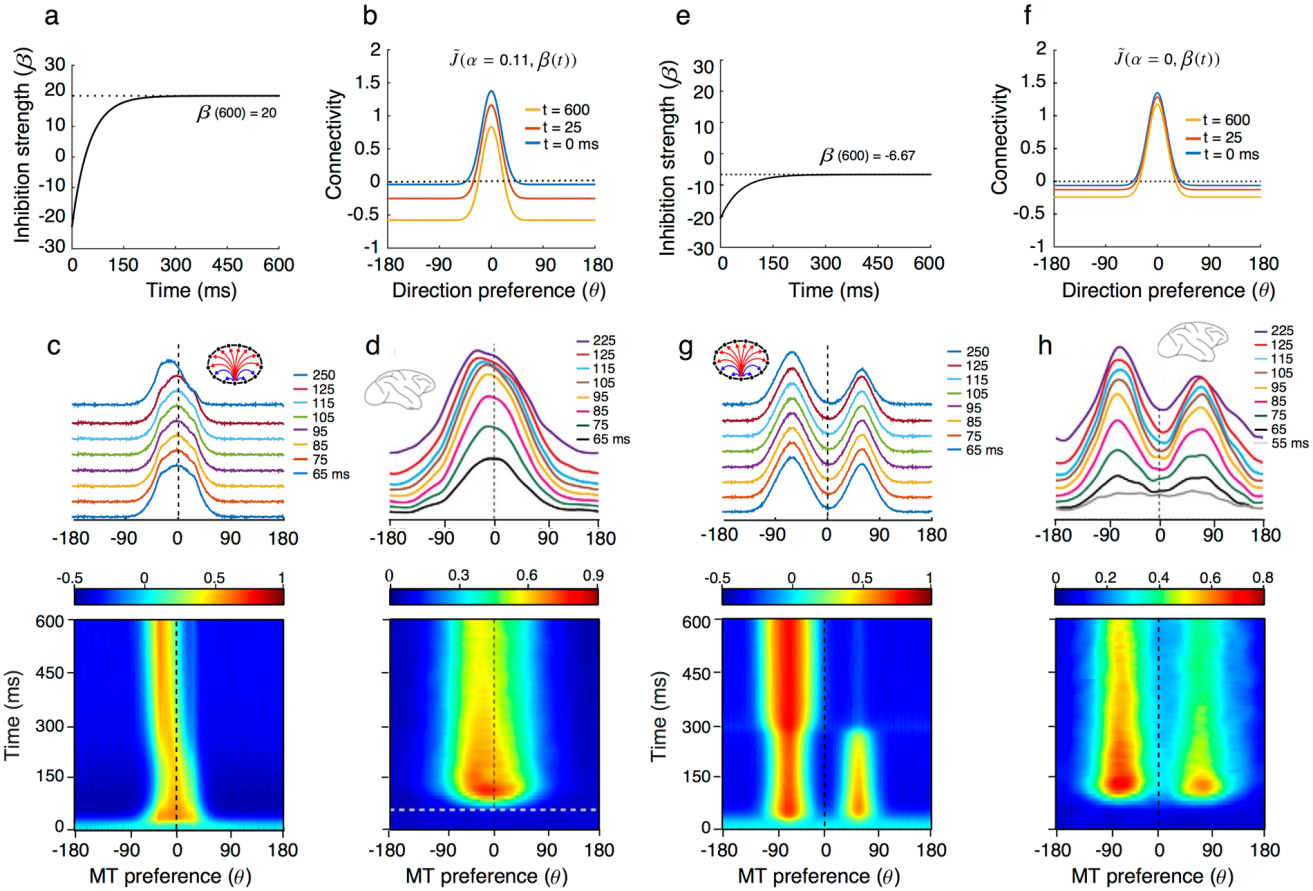
*Temporal tuning behavior*. **(a**–**d)** Temporal development of the WTA solution. **(a)** Time course of inhibition, leading to different excitation/inhibition balance levels as illustrated at three different time lapses **(b)**. **(c)** Temporal evolution of population tuning in response to bidirectional stimuli with PS = 60°. The model population response is initially aligned with the VA direction, before slowly drifting to one of the component directions. The other motion direction is strongly suppressed, corresponding to the WTA solution illustrated by the direction tuning at different time points. The model dynamics renders the temporal evolution of an MT subpopulation as illustrated in **(d)**. **(e**–**g)** Temporal development of neuronal tuning, shifting from TP to SB solution. Same plots as in the left-hand column. Biological data are taken from ref. 10.

We tested this hypothesis by allowing inhibition strength (i.e. β parameter) to evolve temporally with a slow time constant (100 ms). Figure 5 illustrates both model parameters and outputs for two instances of the temporal evolutions shown in Fig. 4a–d. The left-hand column corresponds to the VA-WTA transition. The right-hand column illustrates the time course of the TP-SB transition. Model and MT population^10^ tuning dynamics are illustrated as both full response direction evolution plots and a selection of time slices for comparison (ring-network and macaque brains symbols). Notice that the pure feed-forward latency delaying the MT response by about 50 ms was not simulated in the model dynamics. Upper panels show the time course of surround inhibition together with 3 time-lapses of the excitation/inhibition balance within the direction domain (Fig. 5a,b,e,f). The gradual increase of inhibition strength captures the temporal dynamics of MT motion direction processing. The left-hand panels (Fig. 5c) show that the model population first encodes the VA solution. Slowly increasing inhibition shifts the tuning towards a WTA stable solution, together with an increased precision in the direction tuning, nicely simulating the biological data (Fig. 5d). The right-hand columns illustrate another temporal development when *PS* = 135°. The initial response superimposes the two inputs, corresponding to the T/TP cases (Fig. 5g). Over time, one of the two peaks is suppressed while the other is enhanced. Notice that the former is not entirely suppressed such that until 300 ms, the solution corresponds to the SB case. Later on, it entirely disappears and the late solution becomes a complete WTA tuning function, albeit with a broader tuning that was not reported in the biological data. Furthermore, the two peaks are also shifted away one from another, corresponding to the development of motion repulsion^32^ as noted in^10^. Again, the model dynamics capture the main characteristics of the TP/SB transition illustrated by the mean population activity reproduced in Fig. 5h.

Interestingly, our model could not replicate the transition from a broad VA towards a two peak (T/TP) response that was reported by^10^ (see Fig. 4b). There are two potential reasons for this limitation. First, the likelihood of the two peaks solution is very small when the driving inputs are close and sharp (i.e. small *PW*) as in the case of random dot patterns. Therefore, most of the simulated temporal developments under these conditions occurred between VA and WTA solutions. Even when inhibition strength was set to favor the two-peaks steady-state solution, a large amount of noise was then necessary to destabilize the early VA solution and reach the T/TP solution branch. Second, when the two motion components are close (*PS* = 60°), activity shifts from VA to WTA (Fig. 4a) but for larger *PS* (90° and 135°) activity spreads such that one component builds on while the other vanishes^10^. Instead, under these conditions, the dynamics of our model is characterized by a steady activity of both components, failing to eliminate the weakest peak. This later aspect is consistent with perceptual studies on transparent plaids showing that one component is often perceived weaker and assigned to some background motion (e.g. ref. 33).

We tested other possible time courses of the excitation-inhibition balance by varying the three main parameters controlling inhibition: onset timing, time constant (range 10–100 ms) and final strength. Each parameter strongly influences the time course over which the recurrent network can shift from integration to segmentation. A delayed inhibition results in a stronger VA solution and requires both longer and stronger final inhibition strength to reach either one of the two segmentation solutions (WTA or T/TP). A slower inhibition rise gives longer VA activity shifting towards WTA or T/TP, which occurs very slowly (>400 ms) with weak final inhibition strength. With strong final inhibition strength, the transition is to a WTA solution.

### Predicting tuning behavior for different types of bidirectional motion inputs

**Figure 6:**
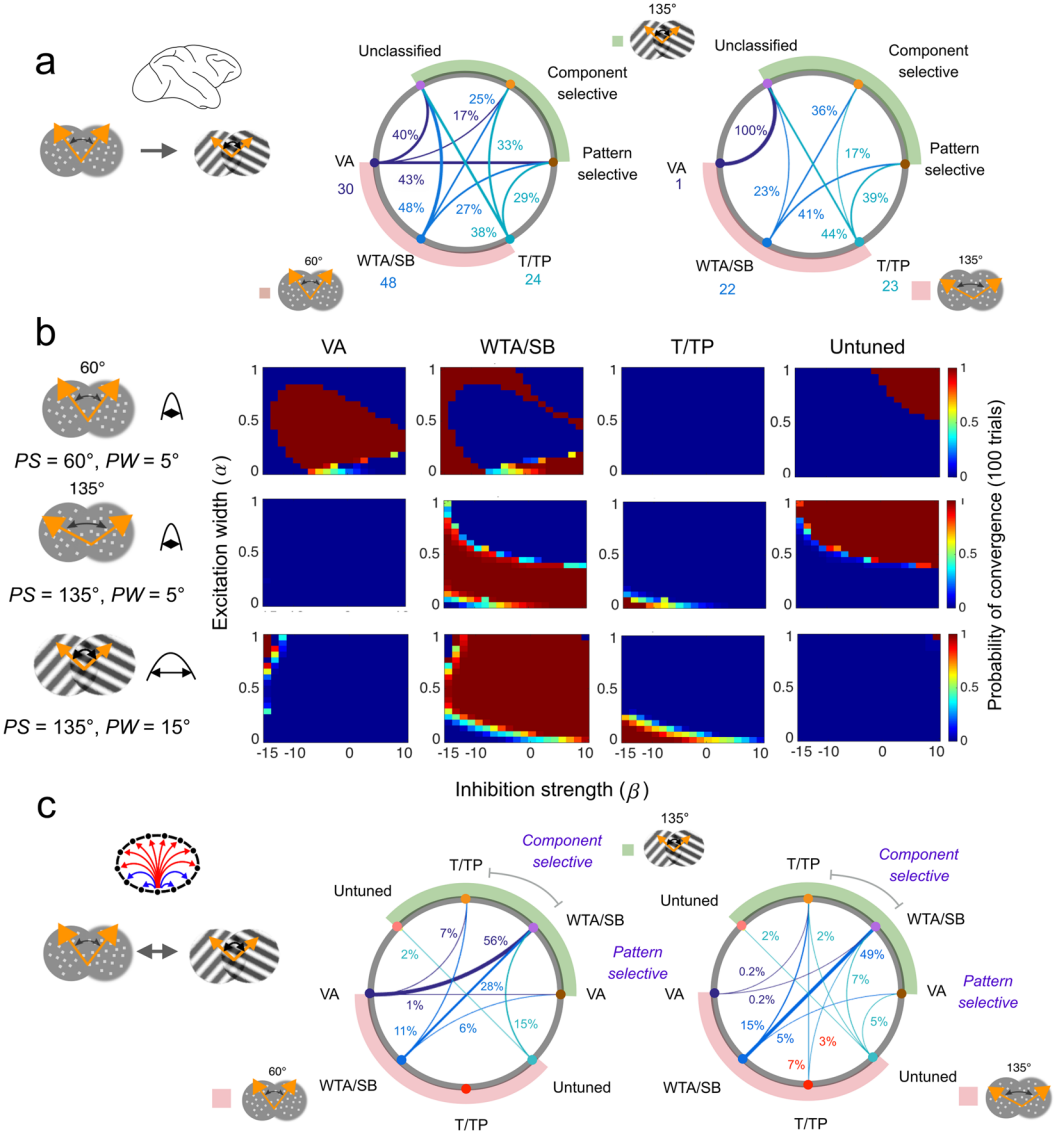
*Responses properties change with input statistics*. **(a)** Direction tuning properties of MT subpopulation shift when presented with either a bidirectional random dot pattern (RDS) or a moving plaid. Migration diagrams have been recomputed from the dataset of ref. 10. For each type of cell obtained with an RDS of *PS* = 60° or *PS* = 135°, the diagram plots their probability of shifting towards either a pattern, a component or an unclassified cell when tested with a plaid pattern of PS = 135°. Overall, the behavior of a subpopulation cannot be predicted when migrating from one stimulus class to another. **(b)** Probability maps of each stable solution (VA, WTA/SB, T/TP, untuned) of the ring model with the same set of bidirectional stimuli, as a function of excitation width and inhibition strength parameters. **(c)** Migration diagrams of the ring model under the same conditions as in **(a)**. The probability of migration from one regime to another is computed from the intersection between pairs of probability maps (see Methods and Supplementary Fig. S1). For the sake of comparison with **(a)**, WTA/SB and T/TP responses to plaids can be regrouped as component selective responses, while VA states can be equated to pattern motion selectivity.

One remarkable property of pattern motion integration in macaque area MT is that cell responses to bidirectional plaid patterns cannot be fully predicted from bidirectional random dot patterns, and reciprocally^10, 11^. That is to say, a pattern cell identified with plaid patterns would not necessarily encode the vector average motion direction of a bidirectional random dot pattern. Xiao and Huang^10^ systematically tested a large population of MT cells with both stimuli, across different *PS* values. From their data (see Table Table 2 in ref. 10), we computed a migration plot illustrating the likelihoods of transition between cell types from one stimulus to another (Fig. 6a). Note that when *PS* = 135° for both plaids and random-dots patterns, the only difference in the input statistics for our model would be *PW* (i.e. the variance in each of the component motion direction provided by the V1 layer). Notice also that in biological studies, unclassified cells are defined only from the component/pattern correlation metrics and such classification is not relevant for our model. Overall, the observed MT migration diagrams show that no strict migration rules were observed but there were rather small biases in how cell tuning migrates when comparing random dots and plaids. Xiao and Huang observed a trend that VA and WTA cells observed with random dots at *PS* = 60° were more likely to be classified as pattern and component cells, respectively when tested with a plaid of *PS* = 60°^10^. When comparing random-dots and plaid patterns with similar *PS* = 135°, T/TP and WTA cells predominantly were classified as pattern selective. Notice that a large fraction of cells that can be classified as either VA, WTA or TP cells in response to random dots were unclassified when tested with plaids, regardless of PS.

In our model, we can simulate these migration probabilities by examining the attractor strength of the stable solutions across a variety of internal parameters allowing us to visualize how the attractor strengths would shift from one condition to another as illustrated in Fig. 6. To do so, we first measured the attractor strength of each solution in different connectivity regimes from 100 responses to a given input as illustrated in Fig. 6b. From these, we constructed a likelihood map for this particular condition. Once the likelihood maps for all the solution types across the different inputs were obtained, we identified the connectivity regimes which could support transitions in one type of tuning for a particular input to other types of tuning with a different input by overlaying the thresholded maps (see the Methods section and Supplementary Fig. 1S) in order to compute an overlap value that illustrates the relative likelihood of a particular migration, as illustrated in Fig. 6c.

When fixing the model internal parameters (i.e. the excitation and inhibition balance) some migrations are more probable than others. With a random dot pattern of small PS (60°), VA and WTA/SB are the most probable states. When presented with a grating of large PS (135°), most VA responses become WTA/SB while WTA/SB responses remains largely identical. When grouping the model response tuning types within the pattern/component classification used for MT neurons with plaids, most of the WTA/SB and VA responses become component selective. Under a fixed inhibition regime, transitions towards pattern selectivity are very sparse. When equating PS between plaids and gratings to 135°, as in ref. 10, the migration patterns become even more dispersed, underlying the critical role of the relative width of motion inputs between the two stimulus types. The majority of WTA/SB responses remained but a significant fraction shifted towards the T/TP states. Only a small fraction of WTA/SB and T/TP responses migrated to VA, as the likelihood of representing pattern motion direction becomes very weak under such large *PS*.

Interestingly, some of the experimentally observed transitions could not be explained by changes in the driving input alone. For instance, the migrations from T/TP to VA reported in both experiments by^10^ (see Fig. 6a) were never observed under fixed recurrent connectivity. Broadening excitation and strengthening inhibition are both necessary to enable these migrations. Moreover, maintaining the VA states across stimulus types would require modulating the excitation/inhibition balance. The grouping of T/TP and WTA/SB states within the classical component selective types of MT neurons (and equating the VA and component selectivity) is somewhat arbitrary. This, together with the limitation that unclassified cell subpopulation was not specifically modeled, prevented us from fitting our model migration patterns to those observed in the neurophysiological studies. Nevertheless, future work with such a ring model could investigate how excitation/inhibition connectivity rules should adaptively change to better predict neuronal tuning changes with input statistics.

## Discussion

A ring-model^27, 34^ with a feature space of motion direction was used to investigate the tuning properties of MT cells in response to inputs with multiple motion directions. We systematically compared our model responses to a recent comprehensive description of MT neuron direction selectivity under a large set of motion inputs^10^. Our simple dynamical model featuring recurrent interactions within MT can produce a surprisingly diverse set of network states that closely match the direction tuning functions associated with different subpopulations in macaque area MT. These tuning functions correspond to the vector averaging (VA), winner-take-all (WTA and its variant, side-bias, SB) or superposition (two-peaks, TP) solutions to the visual motion integration/segmentation computations performed by these neuronal populations (see refs 1 and 4 for reviews). The model shows that these different tuning functions emerge from a single, adaptive recurrent network where the combined effect of the excitation and inhibition could be tuned depending upon on input statistics. Moreover, these recurrent interactions give rise to co-existing network states, that can explain multi-stability and single-unit variability. Recurrent interactions can also explain the dynamics of these tuning functions, i.e. how they transition over time from one type of tuning to another tuning function, and how these subpopulations change behavior when varying the statistical properties of the inputs and the excitation/inhibition regime (e.g., see refs 9–11, 15 and 16). Therefore, our recurrent network model could reconcile a large bulk of recent empirical observations on the properties of area MT neurons, a key area for visual motion processing

### Recurrent interactions allow multiple behaviors from the same network

In our model, individual motion components (e.g., the individual gratings in a motion plaid or, a single dot-field with coherent direction in RDK) were represented by a Gaussian bump in the motion direction space. Inputs with multiple directions were captured by a combination of Gaussian bumps. Varying width and separation of these bumps allows for commonly studied component motion stimuli to be represented in the model. Recurrent connections in the motion-direction space were minimally defined with only two parameters that are the width of excitation and the strength of inhibition. Different network states were characterized in a systematic parameter study varying input statistics and internal connectivity parameters. We have shown that a variety of tuning behaviors observed in the sub-populations of macaque direction selective MT neurons could be explained by a recurrently interacting group of cells tuned to different directions. Previous studies have attributed the different tuning behaviors to functionally different sub-populations^6, 7, 11, 14^. Our model investigates the emergence of these of distinct cell types from the same general network structure based on local differences in recurrent connections combined with asymmetries in the afferent inputs.

Varying the pattern of excitation and inhibition in the direction domain has been previously proposed to simulate these subpopulations in linear-nonlinear feed-forward models (e.g. refs 13, 14 and 35). However, in these feed-forward models, center-surround interactions (in direction space) can only change the weights of different input directions from V1. These interactions are static and fitted to one type of input (e.g. ref. 14 for plaids) and therefore cannot account for all tuning function properties. Only a handful of theoretical studies have proposed that recurrent interactions implementing center-surround interactions in direction space could help solving the motion integration/segmentation problem but they were restricted to implementing a soft winner-take-all mechanism^18, 22, 36, 37^. For the first time, our model captures all currently known tuning properties of macaque MT subpopulations when presented with different types of bidirectional motion patterns (e.g. refs 9–12) and condenses several previous hypotheses on the role (and shape) of inhibition in motion integration and segmentation (e.g. refs 7, 32, 38 and 39). It shows that different sensory tuning functions that are often arbitrarily opposed can be seen as emergent properties of a single recurrent network where excitation/inhibition balance is dependent on the statistical properties of its inputs.

We identified three key input parameters: the direction separation between input distributions, their width and, also, their relative strength. These parameters are well known to control global motion perception and in particular whether the two inputs will be integrated, segregated, repulsed or perceived as transparent surfaces (see refs 1, 2, 39 and 40 for reviews). Given the strong links between the different neuronal tuning functions and each of these percepts, our model could be extended with a simple decoding stage to account for human perceptual performance^37^. Recurrent networks have previously been proposed as a solution for solving some aspects of the integration/segmentation problem (e.g. refs 18, 37, 41–43) and its associated perceptual multi-stability^29, 42^. Because it can reproduce all MT subpopulations and their dynamics across a generic description of the inputs, the current ring model is a major step toward establishing the connection between low-level neural dynamics and canonical computations associated with perceptual dynamics.

### Context dependence of recurrent interactions

An intriguing and controversial property of MT neurons is that, as in many other sensory areas, their direction tuning would depend upon the detailed statistical properties of the inputs^10, 11, 24, 44^. In a recent study, Xiao and Huang^10^ claimed that no simple rule can be used to predict how a vector averaging cell identified with a random dot pattern would behave when presented with a plaid pattern with the same motion component direction. Only some trends could be identified such as more VA and WTA/SB cells would become pattern and component cells, respectively. However, even such a trend is contradicted in another study that reported the opposite migration pattern in the marmoset monkey^11^. These changes in the behavior could be attributed to the differences in the spatio-temporal inputs (in particular the width of each input) as well as by contextual network modulations in terms of lateral excitation and inhibition strength. Our model provides, for the first time, a systemic method to investigate the role of each of these factors.

We reported some of the migration patterns described in macaque area MT^10^ and have shown that a single network (with fixed connectivity parameters) can dramatically change its behavior when shifting from one input to another of similar global and component motion directions but different precisions ﻿(widths). However, the simulated proportions did not fully agree with the experimental data of ref 10. Our results would suggest that the experimentally observed transitions with random dots and plaids are contingent on context-dependent changes for recurrent interactions. For instance, the excitation/inhibition balance might be different for random dot stimuli and for plaids. Further work is needed to unveil the systematic dependence of the recurrent interactions that are needed to predict how MT populations adaptively migrate from one tuning to another. To do so, our qualitative approach would be improved with a systematic fitting of the model parameters to a representative dataset. More important, our study strongly suggests the existence of a context-dependent modulation of the network connectivity parameters that would depend on the type of stimulus, an explanation that reconciles opposite views on the motion integration/segmentation problem.

### A functional consequence of network multi-stability?

Our ring model relies on only four critical parameters in the direction space: the properties of the motion inputs and the center-surround interactions. Our systematic parameter investigation maps the organization of parameter regimes where each of the solutions is stable. A bifurcation analysis revealed regions of the parameter space where multiple network states are possible, leading to multi-stability without any specific plasticity mechanism such as adaptation^23^. To further characterize the different behaviors of the network, we ran stochastic simulations with randomized initial conditions to obtain a measure of the strength of the different stable solutions. This links multi-stability and fluctuations observed at the single-cell level across trials. We found for instance that when the two inputs are symmetrical, we obtained a lack of specific tuning behavior and high-variance in single-cell responses. This high variance only introduces some minor fluctuations in the population activity. This result opens the door to several predictions that could be tested theoretically. First, it would be important to compare population tuning with single cell behavior by contrasting population and unitary recordings. Second, large variability in single cell tuning could be due to network multi-stability. Recently, Ponce-Alvarez and colleagues^21^ demonstrated that rate variability and noise correlation in MT neurons can be seen as emergent properties of a stochastic recurrent network for a set of connectivity parameters that overlaps with a single-state solution and multi-stability. However, their model could not explain the rich temporal evolution of neural response statistics. A further stochastic extension of our ring-model would allow one to render the response variability over the same set of complex stimuli in order to understand how network multi-stability and neuronal variability are related. It would also call for a re-examination of the large proportion of cells that are unclassified in the different empirical classifications that have been used so far in visual studies (e.g. refs 6 and 17). Third, we observed that a small asymmetry in the inputs seems to be critical to stabilize some network solutions and reduce the single-cell variability. This may be critical in natural conditions where small imbalances in the afferent V1 component inputs pooled by MT are highly probable due to direction encoding variability^21^.

### Recurrent models capture slow and fast temporal dynamics of sensory processing

One of the strengths of recurrent network modeling, when compared with the classical feed-forward approach, is its ability to simulate the dynamics of sensory processing. Phenomenological models, often geared towards reproducing psychophysical observations, can capture dynamical characteristics of perceptual competition on slow timescales (generally >10 s). These models are either posed in a discrete (e.g. refs 33, 45 and 46) or a continuous setting (e.g. refs 29, 42 and 47) and have typically focused on dynamical changes in perceived global motion direction or surface motion organization over seconds after stimulus onset. We have previously studied both slow and fast dynamics of motion integration in a continuous recurrent network featuring direction space. Our model can explain many aspects of competitive integration of motion signal such as early vector averaging, transition to one component direction and multi-stability between the different component directions^29, 42^. However, we did not consider motion transparency. Indeed, in our previous model as in nearly all of the existing models, the recurrent network operated prominently in a soft winner-take-all mode^29, 33, 37^. The different tuning behaviors in the current ring model would thus allow investigation of, not only early temporal dynamics, but also population dynamics over slow timescales leading to perceptual shifts between the pattern and transparency perceptual interpretations. We have also shown here that the ring model can render the fast, initial dynamics of motion processing without postulating some specific delays or temporal sensitivities as in previous works^28, 35, 36, 41^. Rather, the time course of inhibition is sufficient to capture the main temporal development of MT direction tuning^15, 16^ as well as the temporal migration from one computation (e.g., VA) to another (e.g., WTA/SB)^9, 10^. This could further explain similar transitions from initial integration to later segregation, which have been observed in both auditory and visual paradigms at the perceptual^48^ and physiological levels^49, 50^. This simple mechanism can easily account for other temporal aspects of MT pattern/component cells such as their temporal integration window^51^ or their responses to triplaids^52^.

Thus, contrary to all previous models, the current ring network can account for all network states associated with the different perceptual interpretations and can bridge slow and fast dynamics of motion integration and segmentation. Moreover, it raises several predictions that can be tested both empirically and theoretically. First, the tuning properties of particular cells would depend upon inhibition strength. Manipulating excitation-inhibition balance is now made possible with new optogenetic tools (e.g. ref. 53). Strengthening surround inhibition should change the temporal dynamics of motion segmentation as well as change the probability of migration patterns when shifting from plaids to random dot patterns. At the theoretical level, the ring model allows for adjustments of excitation-inhibition balance to obtain both of these dynamic properties, something not possible with the existing feed-forward model that requires a set of excitation-inhibition weights for each tuning function^14^. Second, motion transparency can be coded by either neurons with two-peak tuning or by multiplexing of WTA/SB neurons tuned for either one or the other components. Our model predicts that either of these neuronal population codes can be found depending upon inhibition strength and input statistics. Recording MT neuronal populations during perceptual transparent and non-transparent conditions would allow for the versatility of such coding to be tested. At the theoretical level, all previous models worked in a soft-WTA regime and therefore eliminated the TP solutions. The current ring-model predicts that two different inhibition regimes can lead to perception of motion transparency. Third, we found that when the two inputs have the exact same strength, competition yields across-trial fluctuations within the population tuning. Stabilizing the network can be achieved by introducing a small difference between the two components. One consequence is that empirically increasing component contrast or signal-to-noise ratio should affect both the proportion of untuned cells when tested with plaids and the reliability of neuronal tuning of pattern cells in macaque area MT^21^. Previous theoretical or physiological studies have not investigated the role of relative input strength in segmentation/integration dynamics. Input normalization of the classical feed-forward models would prevent investigating the role of relative contrast. The ring-model can explore such perceptually relevant parameters and unveil how it impacts both network stability and the probability of observing different solutions.

### Limits of the recurrent model

The neural field equation describes neural activity in terms of a membrane potential, which is transformed into a firing rate through a sigmoidal nonlinearity. By incorporating additive noise in the model (see ref. 54 for a derivation), some of the variability inherent in a spiking neural network (e.g. ref. 23) is captured despite the fact that a direct link between parameters controlling stochastic properties in spiking and neural field models is not available. However, we expect that our extensive parameters investigation with the current nonlinear model with additive noise has captured the possible dynamics that will be observed with a spiking network. A direct comparison with a spiking network linked to our current model could be done in future work, starting from a more rigorous mean-field description that can capture both the mean firing rate and its variance^55^.

The recurrent network provides a more comprehensive description of motion integration and segmentation in primate visual systems than feed-forward models. In particular, it can qualitatively account for the shift in response tuning properties when shifting from plaids to random dot patterns. To our knowledge, our model is the first to simulate these important aspects. Nevertheless, at present, it fails to capture some aspects of the observed dynamics in macaque area MT. First, we did not observe the transition between broad VA and T/TP solutions as observed by Xiao and Huang^10^ when shifting from plaids to dot patterns. This is explained by the low probability of observing T/TP across a wide range of inputs and the long temporal integration needed to reach this solution. At short time scales, segmentation is dominated by the WTA solution and its variants (SB). Noise properties were constant across the different types of inputs, a characteristic that may not be realistic. A more extensive investigation of the interplay between noise and inhibition properties would unveil the details of the clustering between different subpopulations as characterized by different temporal dynamics when tested with plaid motion inputs^15, 16^. Introducing input modulated noise could impact excitation-inhibition balance and help better simulate the migration patterns observed by Xiao and Huang^10^. Finally, simulating the complex properties of the V1 processing stage (i.e. variability for either random dots or plaid patterns) would improve model performance and better account for the exact migration patterns observed in area MT.

## Conclusion

We have shown that recurrent local interactions in the feature domain can reproduce a variety of tuning behaviors that have been reported in the literature. A single ring network model with direction space center-surround connectivity can capture all of the prototypical tuning curves observed in motion area MT. Interestingly, the attraction strength of different stable solutions and the delayed onset of inhibition, can explain dynamic changes in tuning behavior observed in physiological experiments. These recurrent interactions can form a bridge between models of motion integration and those that capture transparency since they eliminate the need for different sub-populations and strong competition. Unveiling ring attractor dynamics offers a powerful computational framework to simulate adaptive neural computations and paves the way for investigating their physiological implementations across a broad range of neural systems^56^.

## Methods

### Model description

We studied the behavior of direction tuned MT neurons using a continuous ring network model. $u(\theta ,t)\,$defines the activity of the neurons tuned to motion direction $\theta \in [-\pi ,\pi ]\,$and the population dynamics is given by the following neural field equation:

1 $$\frac{du(\theta ,t)}{dt}=-u(\theta ,t){\int }_{-\pi }^{\pi }J(\theta -\varphi )S(\mu u(\varphi ,t),th)d\varphi +{k}_{i}{I}_{ext}(\theta )$$

where, $J$ is the connectivity kernel defined as a weighted difference of Gaussians (DoG) ${J}_{{g}_{e},{\sigma }_{e},{g}_{i},{\sigma }_{i}}(\theta )={g}_{e}G(\theta ,{\sigma }_{e})-{g}_{i}G(\theta ,{\sigma }_{i})$ with *G*(*θ*, *σ*) a Gaussian function, ${g}_{e}$ the excitatory strength, ${\sigma }_{e}$ the extent of the excitatory surround, ${g}_{i}$ the inhibitory strength and ${\sigma }_{i}$ the extent of the inhibitory surround. ${I}_{ext}$ is the driving input corresponding to a particular motion stimulus. $S$ is a sigmoid function where parameter $\mu$ controls the sigmoidal gain. It is defined by the following equation:

2 $$S(\mu u,th)=\frac{1}{1+{e}^{-\mu u+th}}-\frac{1}{1+{e}^{th}},\quad \mu ,th > 0$$

### Representation of visual stimuli using *I*_*ext*_

*I*_*ext*_ is defined as a linear combination of Gaussian bumps, each one corresponding to one component motion direction. The peak width (*PW*) is defined as the standard deviation of the Gaussian bump and renders the inherent uncertainty of the local motion estimation at the V1 stage that feeds the MT layer. The peak separation (*PS*) describes the difference in the motion direction of the components (see Fig. 1b). These variables allow for a complete description of the two main classes of uni- and bi-directional motion inputs used in empirical studies: gratings and plaids on one hand and random-dot patterns on the other. It is then possible to compare the model dynamics across different widely used motion inputs.

### Numerical study of the model

#### Exploration of the connectivity space with ${\tilde{J}}_{\alpha ,\beta }$

The impact of the main parameters governing the nature of local recurrent interactions, the extent of lateral excitation and strength of lateral inhibition were studied using a family of weighted DoG kernels ${\tilde{J}}_{\alpha ,\beta }(\theta )={J}_{{g}_{{e}_{\alpha }},{\sigma }_{{e}_{\alpha }},{g}_{{i}_{\alpha }}+\beta ,{\sigma }_{i}}(\theta )$. More specifically, we focused on the case of uniform lateral inhibition and therefore ${\sigma }_{i}$ is fixed to a large value (${\sigma }_{i}=10\pi$). $\alpha$ is a parameter that smoothly varies the extent of excitatory surround from a narrow ${\sigma }_{{e}_{a}}\,$to a broad ${\sigma }_{{e}_{b}}$ bump, following the equation:

3 $${\sigma }_{{e}_{\alpha }}={\sigma }_{{e}_{a}}+\alpha ({\sigma }_{{e}_{b}}-{\sigma }_{{e}_{a}})$$

The other parameters describing the difference of Gaussian functions, ${g}_{{e}_{\alpha }}\,$and ${g}_{{i}_{\alpha }}$ are estimated in a closed form with the constraint that the first two Fourier coefficients of ${\mathop{J}\limits^{ \sim }}_{\alpha ,0}$ are ${F(\mathop{J}\limits^{ \sim }}_{\alpha ,0})[0]=-1$ and $F({\mathop{J}\limits^{ \sim }}_{\alpha ,0})[1]=1$, which gives:

4 $${g}_{{e}_{\alpha }}={e}^{\frac{-{\sigma }_{{e}_{\alpha }}^{2}}{2}}$$

5 $${g}_{{i}_{\alpha }}=\frac{1+{g}_{{e}_{\alpha }}}{0.0797}$$

$\beta$ is a free parameter to regulate the strength of lateral inhibition. While considering slow evolution of the inhibition, the inhibition strength ${g}_{{i}_{\alpha }}$ varies with time *t* as follows:

6 $${g}_{i}(t)={g}_{{i}_{low}}+({g}_{{i}_{\alpha }}+\beta -{g}_{{i}_{low}})(1-exp\frac{-t}{{\tau }_{l}})$$

#### Parameter regimes supporting transitions

The parametric area supporting a particular transition of interest across different types of inputs (plaids vs. random-dot patterns) is measured by identifying overlapping regimes that support the respective solutions such as VA, WTA or TP. These computing steps are illustrated in the supplementary Fig. S1. In brief, we repeated 100 trials for each input configuration in order to estimate the convergence to each of the solution types. The obtained convergence map was then made binary in order to identify the regimes in which a given solution type is supported. The intersection area of the obtained binary maps would indicate the parameter space supporting the transition of interest. The intersection area relative to the full parameter space was defined as the transition percentage shown in Supplementary Fig. S1.

#### Population versus single unit tuning

In physiological experiments, directional selectivity is measured by sequentially presenting stimuli moving in different directions in a random order. Single cell tuning curves are then constructed by averaging responses across trials. Population tuning is often obtained by aggregating tuning across many neurons from either a single subgroup or the complete sample. Population tuning obtained by different means (e.g., Local Field Potentials) is rarely compared to single cells/subpopulation selectivity, albeit with rare exceptions^57^. Such motion direction selectivity are expressed either in terms of the directional preference of MT neurons or in terms of stimulus motion direction, often assuming that these two dimensions are interchangeable^9^. In a ring model, we can construct a joint representation (matrix) of population versus single unit tuning by sequentially assimilating the population tuning to a stimuli at every motion direction within 0 to 360° along the columns allowing us also to sample the single cell preference. As illustrated in Fig. 2(b0–b4), each column (or vertical cut) shows the population tuning whereas each row (or horizontal cut) shows the single cell tuning. It is important to notice that assuming recurrent interactions between cells tuned to different directions, the population tuning and single cell selectivity are interchangeable only when the network is operating in a regime with a strong attractor bias.

#### Parameter values, initial conditions and numerical computations

The parameters used for the numerical simulations are displayed in Table 1. In case of simulations without noise, standard ode solver, *ODE23T* was used with absolute tolerance value set to 10^−12^. For the simulations with noise, the Euler Maruyama method was used. Initial conditions were set to a low level of random activity. In order to carry out numerical continuation and bifurcation analysis, *Auto07p* package was used, allowing us to track the bifurcation point in one and two-dimensional parameter space. For bifurcation analysis computations were carried out in the absence of noise for a variety of combination of driving input and connectivity kernels. The continuous feature space was discretized into 404 samples.

## Electronic supplementary material

Supplementary Information
