# Supplementary material for: Recurrent network dynamics reconciles visual motion segmentation and integration
Source: Sci Rep. 2017 Sep 12;7:11270. doi: 10.1038/s41598-017-11373-z (PMC5595847; doi:10.1038/s41598-017-11373-z)
Supplement: Supplementary file 1 — Supplementary Information [file 41598_2017_11373_MOESM1_ESM.pdf]

Supplementary Information

Recurrent network dynamics reconciles visual motion segmentation and integration

N.V. Kartheek Medathati [1], James Rankin [2,3], Andrew I. Meso [4,5], Pierre Kornprobst [1] and Guillaume S. Masson [4]

Université Côte d'Azur, Inria, Biovision team, France[1]; Department of Mathematics, University of Exeter, UK [2]; Center for Neural Science, New York University, USA [3]; Institut de Neurosciences de la Timone, CNRS and Aix-Marseille Université, France [4]; Psychology, Faculty of Science and Technology, Bournemouth University, UK [5]

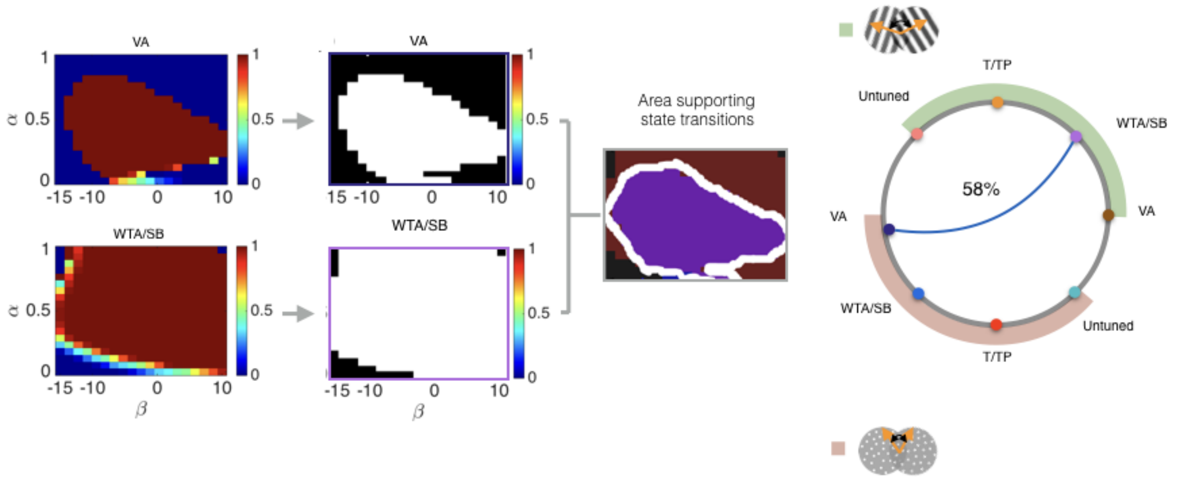

Supplementary Figure 1 (S1). Workflow for computing the percentage of parameter space that supports transitions in the tuning behavior. For the example inputs bidirectional random dot patterns and plaids, we first computed the parameter space supporting a particular tuning such as WTA/SB and VA, respectively. We then converted the convergence probability map obtained using 100 repeated trials in a binary format. In order to measure the space that supports a particular transition, the two binary maps are superimposed to find the area of intersection. The ratio between the intersection and total areas given the frequency of the WTA/SB to/from VA migration reported in the migration diagram on the right-hand column.
